# Assessment of Finite and Infinite Dose In Vitro Experiments in Transdermal Drug Delivery

**DOI:** 10.3390/pharmaceutics13030364

**Published:** 2021-03-10

**Authors:** Luisa Coderch, Ilaria Collini, Victor Carrer, Clara Barba, Cristina Alonso

**Affiliations:** Surfactants and Nanobiotechnology Department, IQAC-CSIC, Jordi Girona 18-26, 08034 Barcelona, Spain; ilariacollini77@gmail.com (I.C.); lcnesl@iqac.csic.es (V.C.); clara.barba@iqac.csic.es (C.B.); cristina.alonso@iqac.csic.es (C.A.)

**Keywords:** in vitro, in silico, permeation/infinite doses, penetration/finite doses, porcine skin

## Abstract

Penetration, usually with finite dosing, provides data about the total active amount in the skin and permeation, being the most used methodology, usually with infinite dosing, leads to data about pharmacokinetic parameters. The main objective of this work is to assess if results from permeation, most of them at finite dose, may be equivalent to those from penetration usually at infinite dose. The transdermal behavior of four drugs with different physicochemical properties (diclofenac sodium, ibuprofen, lidocaine, and caffeine) was studied using penetration/finite and kinetic permeation/infinite dose systems using vertical Franz diffusion cells to determine the relationships between permeation and penetration profiles. Good correlation of these two in vitro assays is difficult to find; the influence of their dosage and the proportion of different ionized/unionized compounds due to the pH of the skin layers was demonstrated. Finite and infinite dose regimens have different applications in transdermal delivery. Each approach presents its own advantages and challenges. Pharmaceutical industries are not always clear about the method and the dose to use to determine transdermal drug delivery. Being aware that this study presents results for four actives with different physicochemical properties, it can be concluded that the permeation/infinite results could not be always extrapolated to those of penetration/finite. Differences in hydrophilicity and ionization of drugs can significantly influence the lack of equivalence between the two methodologies. Further investigations in this field are still needed to study the correlation of the two methodologies and the main properties of the drugs that should be taken into account.

## 1. Introduction

An enormous variety of experimental designs and evaluation procedures for in vitro experiments can be found in the literature. A summary of the most typical finite and infinite dose assays performed in fundamental research is given below. It is important to define a few terms. The amount of active pharmaceutical ingredient (API) that has crossed the barrier is called “the amount permeated”. The amount of substance present in the skin is “the amount penetrated” [1]. In vitro permeation experiments are generally performed using diffusion cells, such as Franz diffusion cells. After sampling, the acceptor solution at different predetermined time intervals and assessed for the cumulative amount that has leached out is evaluated [2]. The amount of permeant in the barrier itself is usually not determined. In vitro penetration experiments can also use Franz diffusion cells; however, the data obtained are not much related to the flux through the skin and drug permeation but mainly related to the drug concentration in the different skin strata. A direct correlation between the penetration data and the permeation parameters has sometimes been found [3], since the diffusion through the stratum corneum (SC) is the rate limiting step.

Both permeation and penetration experiments can be conducted in two scenarios according to the amount of actives applied to the skin, infinite and finite dosage. For infinite dosing, the applied dose is so large that descent of the permeant into the donor chamber or its diffusion into the barrier is negligible. Therefore, the dose could be considered constant and infinite [2]. These large volumes may exert an occlusive effect and thus lead to increased permeability, In the finite dose regimen, only a reduced amount of the donor formulation is applied to the skin, which best resembles the in vivo situation; however, the influence of excipient evaporation can be observed. In the risk assessment of topical products, such as sunscreens, experiments should be performed under real “use conditions” if possible [4].

The usefulness of finite and infinite dose permeation methodologies across human skin was compared. The finite dose model results are much closer to the in vivo absorption data, while the infinite dose results are 10 times higher than the in vivo results [5]. However, it is important to have in mind the most widespread use of the diffusion/permeation infinite dose technique [6,7]. Disagreement between the diffusion coefficient and flux derived from finite and infinite dose experiments demonstrated the apparent alteration in skin barrier properties observed in the infinite dose experiments [8]. Chen et al. found that the total amount of hydrophilic and hydrophobic compounds delivered to the skin significantly increased when an infinite dose was applied but to a significantly different extent [9]. The increase in SC micro-structure modification caused by an infinite dose might not have the same promoting effect for drugs of different hydrophilicity [9]. A model has to be chosen that most accurately fits the consumer use situation. The results from infinite dose experiments served the primary intended purpose of characterizing the compound. Moreover, these parameters could be incorporated into the appropriate equation to allow prediction of anticipated exposure under finite conditions.

On the other hand, there exist numerous Quantitative Structure–Activity Relationship (QSAR) models to predict the permeability coefficient or flux of permeants which may be used to predict the cumulative amount permeated using infinite dosing [10,11]. In the present paper, the Potts and Guy equation [12] which considers the molecular weight (MW) and the solute octanol–water partition coefficient (K_ow_), were applied to be related with the finite and infinite dose in vitro experiments. Other studies attempted to predict finite dose absorption from parameters that can be derived from basic molecular properties of compounds [1]. However, extrapolation of finite dose absorption from parameters estimated with an infinite dose remains a challenge. A very recent work attempted a mathematical approach for predicting skin permeation from a complex vehicle-based formulations applied in finite dose [13]. However, only in a single skin absorption study successful prediction was achieved in which corrections for the ionization state of the permeant were included [14]. This finding indicates that at least for penetration experiments with a finite dose, the ionization state must be considered.

The importance of ionization in transdermal penetration has been studied with benzoic acid [15]. Partition and permeation experiments with human skin indicated that only the undissociated species of benzoic acid is transported through the skin [15]. The ionized form of the compound is minimally soluble within the stratum corneum [16]. When the permeant substance is present in an aqueous solution both as a non-ionic as well as ionic species, the non-ionic (more lipophilic) form is definitely more permeable through the skin. Therefore, skin permeability is highly pH-dependent [16]. The contribution of ionized or unionized species in the total permeation of indomethacin has been also demonstrated [17].

Pharmaceutical industries are not always clear about the method and the dose to use to determine transdermal drug delivery. In transdermal studies, two main in vitro systems are widely used to describe drug transport. Penetration usually with finite dosing provides data about the drug concentration in different skin layers and the total amount detectable in the skin after different incubation times. Permeation, usually with infinite dosing, leads to data that provides information about pharmacokinetic parameters such as the steady-state flux, permeability constant and diffusion rate [3]. When it is necessary to know the amount absorbed in the different layers of the skin, the real quantities of finite doses are chosen, but to compare the penetration of various active ingredients through the skin, infinite dosage is required to evaluate the enough amount in the receptor fluid. Thus, it could be questioned whether it is possible to establish a relationship between the two systems. In addition, the majority of quantitative structure–penetration relationships have been developed to predict only permeability coefficients, maximum flux values and other “steady-state” endpoints based on the results of permeability experiments with infinite dosing.

Therefore, the main objective of this work is to assess if results from permeation, most of them at finite dose, may be equivalent to those from penetration usually at infinite dose. Then, the present work studied the transdermal behaviour of four drugs with different physicochemical properties using penetration/finite and permeation/infinite dose systems. The results were also related to the Quantitative Structure–Property Relationship QSPRs, which are normally used to predict chemical absorption into and through the skin. Interrelation between permeation and penetration data considering the ionization of the different active ingredients in the different skin strata studied was sought.

## 2. Materials and Methods

### 2.1. Materials

Diclofenac sodium (DS), ibuprofen (IBU), lidocaine (LIDO), caffeine (CAF), propylene glycol (PG), and phosphate buffered saline (PBS, pH 7.6) were obtained from Sigma (Sigma-Aldrich, St. Louis, MO, USA). All the buffers and solvents used for practical experiments and the HPLC -quality eluents were purchased from Merck (Merck KGaA, Darmstadt, Germany). The following solutions were prepared in propylene glycol for both assay testing: DS 3% (*w/v*), IBU 1% (*w/v*), LIDO 2% (*w/v*), and CAF 1% (*w/v*). PG was chosen as a solvent because of its great solubility for the different active ingredients, and concentrations were chosen according to those commercially used. Their respective distribution coefficients (LogP) and their molecular weights were calculated in silico using the Pipeline Pilot software (Pipeline Pilot Server 2016 version, Accelrys, San Diego, CA, USA).

A porcine skin membrane was employed for both the permeation and penetration assays. The Institutional Review Board and Animal Ethics Committee of the University of Barcelona (Barcelona, Spain) approved the animal handling protocol (DMAH 5605 approved on 28 January 2013) in accordance with the Guide for the Care and Use of Laboratory Animals, published by the United States National Institutes of Health [18]. The skin was provided by the Department of Cardiology of the Hospital Clinic (Barcelona, Spain). Dorsal unboiled skin (female white/landrace pigs) was prepared: the bristles were removed (A5 Clipper 78005-500, Oster professional, McMinnville, OR, USA) and dermatomed (Dermatome GA630, Aesculap, Tuttlingen, Germany) at 500 ± 50 μm thick. Thickness was verified with a digital micrometer 40EX (Mahr, Göttingen, Germany). Skin biopsies were cut into discs with 2.5 cm diameter. The skin discs were stored until the experiment (at −20 °C).

### 2.2. In Silico Parameters Using Quantitative Structure Permeability Relationship (QSPRs)

The mathematical model of Potts and Guy was used to predict the skin permeability coefficient (Kp in cm/s). This equation takes into account the molecular weight (MW) and the solute octanol–water partition coefficient (Kow):

Log Kp = 0.71 log P − 0.0061MW − 6.3

Two different software platforms, ChemAxon algorythm (14.7.7 version) (Chemaxon, Budapest, Hungary) and BIOVIA PipelinePilot (2016 version) (Pipeline pilot Server, Accelrys, San Diego, CA, USA) were employed to obtain physicochemical properties of the drugs [19].

### 2.3. Water Permeability by Trans-Epidermal Water Loss (TEWL)

The skin barrier integrity of all skin pieces was verified measuring transepidermal water loss (TEWL) using a Tewameter TM 300 (Courage & Khazaka, Cologne, Germany). TEWL, humidity, and temperature were measured for all the skin discs prior to penetration/permeation studies [20]. Those skin membranes that failed in the skin barrier integrity values (TEWL above than 15 g/h·m^2^, OECD Guidelines for the Testing of Chemicals [21]), were discarded and replaced. After stabilization of the cells for 1 h, the measurement of TEWL, temperature, and humidity of skin surface was carried out for one minute, and the mean value was determined.

### 2.4. Kinetic Permeation Assay with Infinite Dosage Using Vertical Franz Diffusion Cells

The kinetic diffusion assay was conducted using a Vision Microette (Vision G2 V14.1) with an Autosampler and an Autofill Collector (Hanson Research, Chatworths, CA, USA) automated vertical diffusion cell (7 mL acceptor chamber, and surface area of application of 1.77 cm^2^).

The receptor fluid (RF) used was aqueous buffer solution of PBS in the case of DS, IBU, and CAF, and NaH_2_PO_4_ (0.05 M, pH = 7) in the case of LIDO. Receptor fluid was continuously stirred with magnetic beads at 700 rpm to keep the contents of the receptor chamber homogenous. The system was thermostatted at 43 ± 1 °C to provide a skin surface temperature of 32 ± 1 °C.

Once TEWL was measured, 300 μL (infinite dose) of the four active solutions was applied in triplicate to each Franz cell (Hanson Research, Chatworths, CA, USA) and donor compartment was sealed to donor chamber to avoid evaporation. Samples (700 µL) were collected at specific times (15 min, 30 min, 1 h, 2 h, 4 h, 6 h, 10 h, 20 h, and 30 h), and automatically passed into the corresponding vial. The same volume of receptor fluid was automatically replaced with fresh solution at each sampling. The content of receptor samples was diluted in 2 mL graduated flasks and filtered with a 0.45 µm nylon filter (Cameo, Sigma-Aldrich, St Louis, MO, USA) previous analyzed with a high-performance liquid chromatography-diode array detector (HPLC-DAD) (Section 2.4). The kinetics of penetration were determined, simulating the drug elimination in the body.

The cumulative amount of the active pharmaceutical ingredient (API) per surface area (*Qn*, µg/cm^2^) was obtained for each replicate cell to calculate the release of the four APIs. The equation used to determine the cumulative amount of API in the receptor fluid was the following Equation (1) [22]:

(1) $$Qn=\;\frac{Cn\times Vc+\;\sum_{i=1}^{n-1}\left(Ci\times Vs\right)}{A}$$

where *Qn* is the cumulative amount of API released at time n (μg/cm^2^); *Cn* is the concentration of active ingredient in the sample (μg/mL); *Vc* is the volume of the vertical diffusion cell; $\sum_{i=1}^{n-1}\;Ci$ is the sum of the concentrations of API (µg/mL) determined at sampling intervals 1 through *n* − 1; *Vs* is the sample volume and *A* is the surface area of the sample.

The percentage of cumulative amount of API permeated over time was calculated by the following Equation (2):

(2) $$\%\;Release\;API=\;\frac{Qn}{Total\;amount\;of\;API\;applied}\times 100$$

Experimental data of cumulative amount (*Qn* and % Release API) were plotted versus time or its square root ($\sqrt{t}$) to obtain the API absorption and penetration kinetics. Different mathematical model (Zero-order, First-order and Higuchi) [23] were evaluated with the non-linear regression software STATGRAPHICS plus 5 (Statgraphics Technologies, Inc., The Plains, VA, USA). The best-fitting model was selected according to the highest correlation coefficient value (r^2^) and it was possible to calculate other parameters, such as flux (J), the permeability coefficient (Kp), lag time (Tl), the maximum concentration (Cmax), the maximum time (tmax), and the area under the curve (AUC).

### 2.5. Penetration Assay with Finite Dosage Using Vertical Franz Diffusion Cells

OECD guidelines [21,24] and the published opinions of the Scientific Committee on Cosmetic Products and Non-Food Products (SCCS) [25] were adhered in the penetration protocol. The excised pig skin was placed in a static Franz diffusion cell (Lara Spiral, Couternon, France) (1.86 cm^2^ and 3 mL) with the dermis side facing the acceptor chamber. The receptor chamber was filled with receptor fluid. The receptor fluid was the same used in the kinetic assay (Section 2.4). Air bubbles entrapped below the skin were carefully removed and the assembled cells were deposited in a temperature-regulated water bath (Telesystem HP 15, H+P Labortechnik GmgH, Oberschleissheim, Germany) on top of a water-resistant magnetic stirring plate (Variomag 15 and Telemodul, H+P Labortechnik GmgH, Oberschleissheim, Germany). The homogeneity of receptor fluid was maintained by continuously stirring at 700 rpm. The system was thermostatted at 43 ± 1 °C to provide a skin surface temperature of 32 ± 1 °C.

The integrity and permeability of membranes were evaluated with the trans-epidermal water loss (TEWL) value as detailed in Section 2.3. Lidocaine, diclofenac sodium, ibuprofen and caffeine were selected to be compared with the infinite kinetic results. The concentration of solutions of active drugs was the same as of kinetic assay.

The finite assay protocol was conducted as described in previous studies [26]. Briefly, 20 µL of each API solution was applied to the skin surface in the donor chamber (*n* = 3). According to the OECD methodology used [21], penetration studies were performed for 24 h. After the exposure time, the receptor fluid of all the membranes was collected to transfer to a 5 mL volumetric flask. The remaining API solution was removed from skin surface with specific washing procedure (W). Stratum corneum layers were obtained by tape stripping protocol with adhesive tape (D-Squame, Cuderm Corporation, Dallas, TX, USA) applied under controlled pressure (80 g/cm^2^). Viable epidermis (E) and the dermis (D) were separated after heating the skin at 80 °C for several seconds. All samples were extracted with adequate solvent (Table 1) and filtered with a 0.45 µm Nylon filter (Cameo, Sigma-Aldrich, St. Louis, MO, USA) before HPLC quantification (Section 2.6).

The resulting mass balance was acceptable (100 ± 15%) for each compound after an exposure time of 24 h. The results are presented as the normalized amounts (%) of penetrated substance and their standard deviations. The amount permeated in the skin was considered the sum of that in the epidermis, dermis, and receptor fluid [21].

Since permeability constants were obtained by the permeation kinetic assay and a mathematical model, a permeability constant with results from the penetration assay was also calculated for easy comparison. This following equation reported by Lian [27] assumes that the system was at the steady state, even though the system will hardly reach steady state. In addition, the equation underlines that the rate of the substance being transferred across the skin obeys Fick’s law of diffusion.

$$\frac{dM}{dt}=K_{p\;}\cdot A\cdot \left(C_{1}-C_{2}\right)$$

The steady-state flux through the skin (dM dt) was considered as the absorbed amount of substance permeating through the skin (equivalent to the sum of that in the epidermis, dermis, and receptor fluid) divided by the time (24 h). The surface area of exposure, *A*, was 1.86 cm^2^. $C_{1}$ is the concentration of the donor solution, and $C_{2}$ is the receptor fluid concentration.

### 2.6. High Performance Liquid Chromatography with Diode Array Detector (HPLC-DAD) Analysis

All analyses were performed with reverse-phase HPLC, employing a VWR HITACHI ELITE LaChrom instrumentation (VWR International, Labexchange, Burladingen, Germany) equipped with a pump with piston flushing (HTA L-2130), an auto-sampler L-2200, and a diode array detector (DAD-detector CM5430). The software used was EZChrom Elite c 3.1.6.

The amount of active ingredient in the samples was determined by an HPLC methodology validated according to ICH Q2 (R1) guidelines in terms of linearity, accuracy, and precision [28]. Parameters of the calibration curve, the limit of quantification (LoQ), and limit of detection (LoD) were determined. The HPLC-DAD analytical conditions and the isocratic method for the four active ingredients are detailed in Table 1. These conditions were the same for the analyses of the exact concentration of the starting solutions and for the analyses of the samples of the kinetic permeation and percutaneous absorption Franz cell assays (Section 2.4 and 2.5).

### 2.7. Statistics Analysis

The statistical analyses of the cumulative amounts of the API released (infinite dosage study) and penetrated (finite dosage study) were determined using the non-linear regression analysis. All the results are expressed as the mean ± standard deviation (SD).

Permeation parameters as well as penetration results were statistically compared with the non-parametric test of Kruskal–Wallis. Statistical significance was tested at the 0.05 level of probability (*p*).

STATGRAPHICS plus 5 software (Statgraphics Technologies, Inc., The Plains, VA, USA) was used for all statistical evaluation.

## 3. Results

Four drugs with different physicochemical properties were studied using infinite dose assay obtaining a permeation data and using finite dose system obtaining penetrated amount results. The results were also compared with the QSPRs which are normally used to predict chemical absorption into and through the skin. Diclofenac sodium, ibuprofen, lidocaine, and caffeine were chosen due to their wide range of lipophilicity and different molecular weights. Moreover, diclofenac sodium and ibuprofen present an acidic behavior while lidocaine and caffeine present a basic one, as ibuprofen and lidocaine partially unionized at the skin pH. These differences would probably affect the penetration/permeation of the drugs important for the assessment of these methodologies.

### 3.1. In Silico Predictions Using QSPRs

Physicochemical properties were calculated for all drug compounds using the Chem Axon software platform (Table 2). The different physicochemical properties translated into a wide range of skin permeability values, allowing us to make conclusions regarding the link between these active properties and the permeability prediction obtained in the in silico and in vitro models. The skin permeability coefficients (log Kp) were calculated with the Potts and Guy model [12].

**Table 2:** The pKa values, percentage of ionized/unionized compounds, octanol–water distribution coefficients (log P), and molecular weights (MWs) obtained from the ChemAxon platform and in silico permeability log Kp (Potts and Guy).

Compound	pKa	%ion/union pH 5.5	%ion/union pH 7.0	LogP (pH = 7.4)	MW	Log Kp (Potts and Guy)
Diclofenac sodium	4.15 [29]	95.7	99.9 (−)	1.10	318.13	−7.42
Ibuprofen	5.30 [30]	61.2	97.8	3.97	206.3	−4.74
Lidocaine	7.70 [31]	99.9 (+)	85.2	2.44	234.3	−5.99
Caffeine	10.4	100 (+)	100 (+)	−0.07	194.2	−7.53

These four active ingredients demonstrated different physicochemical properties. Their acid-base properties (pKa) indicated the acidic behavior of diclofenac sodium and ibuprofen and the basic behavior of lidocaine and caffeine. Since the surface of the skin has a pH of approximately 5.5 and this pH increases in the inner skin strata to approximately 7 at a systemic or receptor fluid level [32,33], the percentages of ionized and unionized drug for all compounds following the Henderson–Hasselbach equation were calculated at these two pH values for the four drugs [34,35]. While diclofenac sodium and caffeine were mostly ionized in the range of the two pH values studied (negative and positive respectively), ibuprofen and lidocaine were much unionized at pH 5.5 and pH 7.0. According to these data, ibuprofen and lidocaine would be the compounds that would penetrate the most, since skin penetration is favored for unionized compounds [15,16].

In addition, these compounds have a wide range of lipophilicity (expressed as the partition coefficient in octanol/water (log P) and different molecular weights (MWs) (Table 2). The Potts and Guy equation considers the molecular weight (MW) and the solute octanol–water partition coefficient log P.

This model considers the substance lipophilicity, the most important parameter that affects skin permeation. Ibuprofen and lidocaine are the most lipophilic compounds, and they have a higher log P; these values are followed by those for diclofenac sodium and caffeine. Therefore, the rank order of the QSPR predictive model is IBU >> LIDO >> DS ≥ CAF.

### 3.2. In Vitro Permeability Models

According to EMA [36] to evaluate transdermal absorption, human skin is the most relevant membrane. However, its availability is limited. Skin alternatives such as artificially cultured human skin models, parallel artificial membrane permeability assays (Pampa), and artificial membranes have been recently comprehensively discussed [37,38,39] In principle, the use of skin cultures is limited due to lack of effectiveness as a barrier in skin permeation studies, as well as their cost and reproducibility. A wide range of animal models has been suggested, and the most relevant animal model for human skin is pig skin [40].

#### 3.2.1. Infinite Dose Permeation Assay

**Figure 1:**
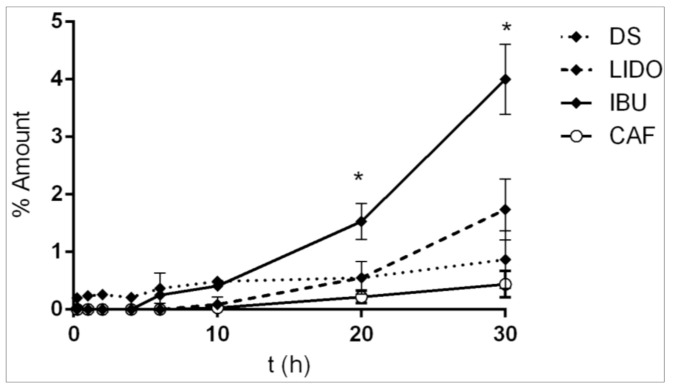
Mean percentage of release of diclofenac sodium, lidocaine, ibuprofen, and caffeine applied at an infinite dose (* indicates significantly different, *p* < 0.05).

The kinetic permeation assays with infinite dosage were performed for the four compounds as described in the experimental Section 2.2. API release was determined through the cumulative amount released (Qn, μg/cm^2^), which is equivalent to the total amount of API quantified in the receptor fluid per unit area [22]. Other kinetic parameters were determined (Flux, C max) as detailed in the experimental section. The percentage of drug released over time was measured for the different four active ingredients obtaining permeation properties of each one. The results are expressed in Table 3 and shown in Figure 1.

The release kinetics on skin for all actives followed a zero-order model. This mathematical model proved the controlled delivery of APIs due to the implication of the skin membrane rather than the amount applied. Some coefficients were directly related to the active concentration, such as J (µg/cm^2^/h), Cmax (µg/mL), and AUC (µg·h/mL). Since the concentration of active ingredients varies depending on their commercial use, the permeability kinetics focused on the Kp and AUC (%).

The rank order of permeation kinetics through the skin (Kp) was IBU >> DS ≥ LIDO >> CAF. IBU has the statistically highest lipophilicity and a very low molecular weight. Moreover, the value of its pKa is 5.30 and it is almost unionized (≈5.50) [32] at the pH of skin. Therefore, ibuprofen had the statistically higher permeation, as expected. In contrast, CAF was the most hydrophilic, even with the lowest molecular weight, and its pKa of 10.4 indicates fully positive ionization of the molecule in the whole range of the pH of the skin. Therefore, the lowest skin permeation occurred with caffeine. An intermediate permeation was observed for the two other active ingredients (Figure 1). The kinetic behaviour of DS somehow differed from those of the other compounds. Its release was higher at short sampling times, but it had been moderated at long times (at 20 h) being lower that of LIDO. DS is an acid (pKa = 4.15) that is partially unionized at the pH of the skin, which leads to high absorption, but its high hydrophilicity and MW promote a delay in penetration at longer times. In this case, the increase in skin pH led to more difficult absorption at longer times.

#### 3.2.2. Finite Dose Penetration Assay

In the percutaneous absorption assay with finite dosage were performed for the four compounds as described in the experimental Section 2.3. Distribution of the selected compounds was determined in the various skin compartments and in the receptor fluid mimicking the systemic system. Therefore, this in vitro system was used to compare the skin penetration profiles of each compound. Thus, it is important to discern that this assay is not a kinetic permeation assay, but it describes the very minor amount of each active ingredient in the different skin strata and in the receptor fluid.

**Figure 2:**
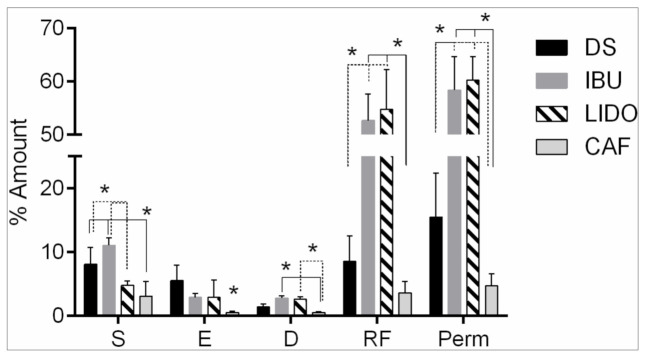
Mean percentages of the amount of diclofenac sodium, ibuprofen, lidocaine, and caffeine applied in finite doses in the different skin strata (stratum corneum (S), epidermis (E), and dermis (D)), receptor fluid (RF), and permeated amount (Perm) (* indicates significantly different, *p* < 0.05).

The results obtained are reported as the amount accumulated after 24 h of skin contact. The amount of each active ingredient in the stratum corneum, other epidermis layers, dermis, and receptor fluid are shown in percentage units to avoid penetration variances caused by different concentrations. Results are indicated in Table 4 and Figure 2. The concentrations retained by the stratum corneum are considered as unabsorbed by the skin and do not contribute to the systemic dose. However, the concentrations found in the epidermis and dermis could be considered absorbed, reaching a systemic level [21]. Therefore, the amount of percutaneous absorption (Perm) is normally assumed to be the sum of the concentrations in the epidermis (E), dermis (D), and receptor fluid (RF). The mass balance is acceptable, being in the range 100 ± 15% for each compound after the exposure time of 24 h

In this case, the rank order of active penetration through the skin was LIDO ≥ IBU >> DS >> CAF. As previously mentioned, caffeine has, as expected, the statistically lowest permeability due to its lowest lipophilicity being fully ionized in the entire skin pH range. Ibuprofen demonstrated very high penetration at 53% and it was the active ingredient present in the highest amount in the stratum corneum, with a percentage of 11%. IBU was mostly unionized at pH 5.5 and much more stable in the skin surface. Lidocaine was the least ionized compound at pH 7, and this could be the reason for the higher amount of the drug in the dermis and in the receptor fluid. Diclofenac sodium had an intermediate permeability, and its partial unionization at pH 5.5 induced a high amount of active ingredient in the stratum corneum and epidermis with small amounts in the dermis and receptor fluid because of its almost negative ionization at pH 7.

## 4. Discussion

From comparison between the in silico model and the two in vitro methodologies, there is a general agreement between the rank order of compounds IBU > DS > CAF; however, permeation of lidocaine varies considerably as it is the one that penetrated the most in the finite dose penetration assay. Lidocaine is practically fully ionized at pH 5 and the least ionized compound at pH 7. The greater influence of the stratum corneum barrier function (pH 5.5) in the permeation assay could account for its lower permeation (in the permeation infinite assay). The influence of the amount absorbed in the epidermis, dermis, and the receptor fluid (pH 7) in the penetration assay could account for its higher penetration.

To better compare permeability differences among active ingredients with the different methodologies, the permeability constants (Kp) from results obtained with this last penetration assay were calculated by the equation reported by Lian [27], assuming the system was at steady state, even though the system will hardly reach steady state, and the flux through the skin was the absorbed amount of percutaneous substance (experimental section). Therefore, the Kp of the penetration/finite assay would not be so reliable than the ones of permeation/infinite dosage.

In general, the results of percutaneous absorption and the corresponding log Kp obtained were in accordance. Due to the acquisition of log Kp, the Franz cell data could be compared with the permeability constants obtained by the kinetic study and the mathematical formula (Table 5). However, the finite doses for the percutaneous absorption assay could be expected to yield lower diffusion coefficients than the permeation kinetic assay with infinite doses and the mathematical models.

The in silico Potts and Guy model, which is based on other authors’ experimental results of kinetic permeation, indicated a very low permeability for caffeine (log Kp −7.53), mainly due to its high hydrophilicity, and quite similar permeability to that of diclofenac sodium (log Kp −7.42), because of its high molecular weight. Lidocaine had higher permeability (log Kp −5.99) due to its lower molecular weight than that of diclofenac sodium and its higher hydrophobicity than those of caffeine and diclofenac sodium. Ibuprofen having the highest permeability (log Kp −4.74) was mainly due to its having the highest hydrophobicity with a very low molecular weight. However, this model does not consider the different permeability values caused by the different pH values in the different skin strata. Despite the numerous QSAR models for predicting permeability [10], and the general knowledge of the influence of ionization on skin permeation, to our knowledge, there are not many studies in which, in addition to the physico-chemical parameters, corrections for the ionization state of permeant, were included [14]. Since lipophilicity is strongly pH-dependent [29], this model was modified to consider the degree of ionization of the active ingredient and the properties of the vehicle [14]. The degree of ionization of the active ingredient was considered for weakly basic and weakly acidic compounds as a function of the vehicle pH.

In the kinetic permeation assay, the lowest value of skin permeation was also for caffeine and the highest for ibuprofen, as in the QSAR model. Intermediate permeation was obtained for lidocaine and diclofenac sodium. However, the theoretically higher permeability for lidocaine due to its high hydrophobicity and low molecular weight was not substantially reflected in the AUC% or Kp, which was similar for the two compounds. The greater amount of unionized compound for diclofenac sodium at the pH of the skin could account for the initial high absorption in the stratum corneum, leading to higher permeation than that theoretically calculated by the Potts and Guy model. The permeability increased as the percentage of ionized/unionized species decreased to pH 5.5 (Table 2). This relationship indicates that in this infinite dose steady/state assay, the stratum corneum plays a relevant role in the total permeability and it is considered the rate limiting step.

As previously mentioned, the minor finite doses for penetration assays result in lower diffusion coefficients than the ones corresponding to the kinetic assay with infinite doses and the mathematical model (Table 5); besides, a lower reliability has to be taken into account due to the probably non steady state of the system. However, as in previous cases, caffeine had the lowest diffusion coefficient (log Kp −8.17). Ibuprofen demonstrated very high permeation (log Kp −7.17), and lidocaine demonstrated even higher permeation (Kp −7.10), which could be due to less ionized compounds at pH 7, the pH of the receptor fluid. The permeability was observed to increase as the percentage of ionized/unionized species decreased towards pH 7. This result indicates that in this finite dose non steady/state assay, there is much influence of the solubilization of the compound in the inner skin layers and in the receptor fluid, which facilitates percutaneous absorption.

Franz et al. [8] predicted the flux of an infinite dose using data obtained from fitting finite dose experiments. The observed flux was considerably greater than that predicted. This finding was interpreted as an indication that due to the large amounts of formulation applied, the barrier function of the skin had been impaired. Moreover, it has been proposed that the hydration effect of an infinite dosage in the stratum corneum could make the penetration of hydrophilic drugs easier than hydrophobic drugs [9]. This could also explain the similar log Kp for the permeation experiments with an increased value for caffeine and diclofenac sodium compared with the theoretical in silico log Kp values and the finite dose results. Moreover, the unmodified microstructure of the SC by a finite dose [9] results in a large difference in penetration among compounds with a much lower log Kp for the hydrophilic compounds, diclofenac sodium, and caffeine.

The majority of studies done to assess prediction of skin permeation were performed with infinite dosage, whereas only a limited number of studies have been conducted for finite dose conditions [13]. Other reseachers examined reliable prediction of penetration results from in vitro infinite dosing permeation results or from physicochemical properties of the active ingredients. Many in vitro predictions aimed to envisage the cumulative amount penetrated, since determination of the amount of permeant in the skin is difficult and usually involves destruction of the barrier. Thus, Seta et al. [41] simulated the concentration−time course in hairless guinea pig skin and the receptor in vitro, and Krüse [42] and Frasch and Barbero [43] extrapolated the time course of a permeant for finite dosing from parameters derived from infinite dose studies, and they found an overprediction of accumulated mass. Buist et al. [44] also obtained an overestimation of the tested drug absorption. Other authors provided Laplace solutions for the amount of drug in the donor and acceptor and within the membrane [45,46,47]. Wagner et al. applied a lipophilic model drug preparation using infinite dose conditions in two cases [3]. The flux data of permeation experiments for an isolated stratum corneum and a separated epidermis were linearly related to the mass amount values of penetration experiments. However, this interrelation between permeation and penetration data was only shown for one lipophilic drug of flufenamic acid in a lipophilic vehicle, in the two cases when infinite dosage was applied. Caution must be considered in extrapolating data derived from infinite/simple vehicles to finite/complex formulations used in practice [13]. Few studies have been performed to predict skin permeation in finite dosage in actual cosmetic formulations [13,14,48]. Arce et al. [13] incorporated evaporation kinetics and vehicle-permeant dependent parameters. Gregoire et al. [14] studied a finite dose absorption prediction using parameters that a scientist can derive from basic molecular properties. Corrections for the ionization state of the permeant were included and the skin absorption of two compounds was successfully predicted. Generally, the predictions yielded unsatisfactory results, especially for long experimental times. Extrapolation of absorption at finite dose from parameters considered in the infinite dose case remains a challenge [1].

From our results, we can conclude that good correlation of these two in vitro permeation-infinite dosage and penetration-finite dosage results is very difficult to find. There is a hydration effect or a modification of the microstructure of the stratum corneum with an infinite dosage, which enhances hydrophilic drug penetration to a higher extent than hydrophobic drug penetration. Moreover, ionization of the active ingredients in the different skin strata seems to play a different role in permeation in the two assays. Permeability increases in the kinetic permeation assay with infinite dosage as the percentage of ion/union decreases at pH5.5 and it increases in the percutaneous absorption assay with finite dosage as the percentage of ionized/unionized decreases at pH 7 (Table 2). These findings indicate that the pH of the different skin strata can play a relevant role in the total permeability favouring the permeation of undissociated species mainly through the stratum corneum in the kinetic permeation assay and through the dermis and fluid receptor in the percutaneous absorption assay.

Finite and infinite dosages have different applications in transdermal delivery which present their own advantages and challenges [49]. Mathematical models allow us to characterize, predict, and compare skin absorption kinetics related to finite and infinite dosing. However, in applying these models, it is important to appreciate the limitations of them. Being aware that this study only presents results for four actives with different physicochemical properties, it can be concluded that the permeation/infinite results could not be always extrapolated to those of penetration/finite. Differences in hydrophilicity and ionization of drugs can significantly influence the lack of equivalence between the two methodologies. Further investigation in this field is still needed to study the correlation of the two methodologies and the main properties of the drugs that should be taken into account.

## 5. Conclusions

Drug transport was evaluated with the two mainly used in vitro systems described in the introduction and methodology sections. Permeation with infinite dosing provides information about steady-state flux, the diffusion constant and permeability. Penetration with finite dosing, evaluates the drug concentration in different skin layers and the total amount of drug in the fluid receptor after 24 h. The aim of this study was to discern the possible relationship of the results using the two methodologies. These results were also related with in silico predictions using a quantitative structure−penetration relationships model. Therefore, the skin transdermal results of the four drugs with different physicochemical properties were studied using infinite dose assay obtaining permeation data and using a finite dose system obtaining penetrated amount results. The results were also compared with the QSPRs which are normally used to predict chemical absorption into and through the skin.

The in silico Potts and Guy model, which is based on experimental results of kinetic permeation, can help to predict the permeation behaviour of compounds. However, this model does not consider the different permeability values due to dosage or different pH values in the different skin strata. The transdermal delivery of four compounds (caffeine, ibuprofen, diclofenac sodium, and lidocaine) was obtained with different in vitro methodologies: (1) infinite application dosage obtaining their release profile and (2) finite dosage determining the amount at different skin strata at 24 h. Good correlation between results from the two methodologies was not always obtained. The hydration effect of infinite dosage in the stratum corneum could facilitate the penetration of hydrophilic drugs in front of hydrophobic drugs. Moreover, the pH of the different skin strata could play a relevant role in the total permeability favouring the permeation of undissociated species in the percutaneous absorption assay. Therefore, similar permeation was observed in the different assays; however, the active ingredient penetration depends not only on their physical properties, such as molecular weight, hydrophilicity, and melting point, but also largely on their dosage and the proportion of different ionized compounds due to the pH of the skin layers, playing a different role according to the chosen permeation test.

Therefore, it can be concluded that the permeation/infinite results could not be always extrapolated to those of penetration/finite. Differences in hydrophilicity and ionization of drugs can significantly influence the lack of equivalence between the two methodologies. Since only four actives have been studied, further investigations in this field are still needed to follow the correlation of the two methodologies and the main properties of the drugs that should be taken into account.

## Figures and Tables

**Table 1:** HPLC analytical conditions and parameters for diclofenac sodium (DS), ibuprofen (IBU), lidocaine (LIDO), and caffeine (CAF).

Parameter	Diclofenac Sodium	Ibuprofen	Lidocaine	Caffeine
Extractor solvent	CH_3_CN (+CF_3_COOH 0.5%)	CH_3_OH	CH_3_OH	CH_3_OH:H_2_O (1:1)
Column	LiChrocart^®^250-4 Lichrosphere^®^ 100RP-18, 5 µm	LiChrocart^®^250-4 Lichrosphere^®^ 100RP-18, 5 µm	LiChrocart^®^ 125-4 Lichrosphere^®^ 100RP-18, 5 µm	LiChrocart^®^ 125-4 Lichrosphere^®^ 100RP-18, 5 µm
Wavelength (nm)	254	221	205	271
Injection volume (µL)	20	40	20	20
Mobil phase (flux)	66% CH_3_OH 34% H_3_PO_4_ 0.7% (1 mL/min)	67% CH_3_OH 33% H_3_PO_4_ 1.2% (1 mL/min)	70% NaH_2_PO_4_, 0.05M pH 7.4 30% CH_3_CN (1 mL/min)	75% CH_3_OH 25% H_2_O (1 mL/min)
Linear regression equation (R^2^)	$A=80050\left[DS\right]-2484$ $\left(0.9997\right)$	$A=305873\left[IBU\right]-23295$ $\left(0.9993\right)$	$A=414046\left[LIDO\right]-68532$ $\left(0.9999\right)$	$A=237268\left[CAF\right]-21113$ $\left(0.9999\right)$
LoD/LoQ (µg/mL)	0.07/0.22	0.18/0.55	0.15/0.31	0.09/0.29
Precision (%CV) Intra day	2.05 ± 0.71	3.25 ± 1.60	5.21 ± 2.86	2.34 ± 1.07
Inter day	6.02 ± 1.98	3.59 ± 0.98	5.30 ± 3.03	2.81 ± 2.07

**Table 3:** Mean values of flux (J), permeability coefficient (Kp), maximum concentration (Cmax), and area under the curve (AUC) for Table 0. (* indicates significantly different, *p* < 0.05; ^a^ indicates significantly different compared to ibuprofen, *p* < 0.05).

Parameter	Diclofenac Sodium 3%	Ibuprofen 1%	Lidocaine 2%	Caffeine 1%
Kin. of release	Ord 0	Ord 0	Ord 0	Ord 0
J (µg/cm^2^/h)	3.94 ± 0.77 *	2.60 ± 0.69	2.29 ± 0.30	0.86 ± 0.07
Kp (cm/h)	1.59 × 10^−4^ ± 2.01 × 10^−4^	2.50 × 10^−4^ ± 6.00 × 10^−5^	1.20 × 10^−4^ ± 4.00 × 10^−5^	0.87 × 10^−4^ ± 1.00 × 10^−5^ *
Cmax(µg/mL)	38.10 ± 10.01 ^a^	66.71 ± 16.06	49.95 ± 11.53	13.88 ± 1.59 *
AUC (µg·h/mL)	167.55 ± 38.54	163.48 ± 28.25	109.30 ± 35.09 *	72.82 ± 32.56 *
AUC (%)	15.43 ± 4.41	38.97 ± 4.56 *	14.95 ± 6.09	4.55 ± 3.31

**Table 4:** Normalized amounts of active ingredients (% API) found in the stratum corneum (SC), epidermis (E), dermis (D), receptor fluid (RF), total permeation (Perm), and log Kp.

% API Detected	Diclofenac Sodium 3%	Ibuprofen 1%	Lidocaine 2%	Caffeine 1%
SC	8.08 ± 2.62	11.06 ± 1.15	4.77 ± 0.70	3.11 ± 2.33
E	5.54 ± 2.41	2.92 ± 0.61	2.92 ± 2.69	0.55 ± 0.23
D	1.41 ± 0.49	2.79 ± 0.37	2.60 ± 0.40	0.55 ± 0.09
RF	8.53 ± 3.99	52.68 ± 5.01	54.73 ± 7.48	3.62 ± 1.75
Perm	15.49 ± 6.89	58.39 ± 6.32	60.25 ± 4.39	4.72 ± 1.87
Mass balance (%)	103.80 ± 9.21	92.81 ± 13.97	90.14 ± 6.65	98.92 ± 1.32
Log Kp (cm/s)	−7.70	−7.17	−7.10	−8.17

**Table 5:** The pKa, log Kp Potts and Guy, log Kp from the experimental infinite dose kinetic assay, and log Kp from the experimental finite dose percutaneous absorption assay.

Compound	pKa	Log Kp In Silico (Potts and Guy)	Log Kp Permeation Infinite Dosage	Log Kp Penetration Finite Dosage
Diclofenac sodium	4.15	−7.42	−3.80	−7.70
Ibuprofen	5.30	−4.74	−3.60	−7.17
Lidocaine	7.70	−5.99	−3.92	−7.10
Caffeine	10.4	−7.53	−4.06	−8.17

## Data Availability

Not applicable.
